# Telomerase inhibition abolishes the tumorigenicity of pediatric ependymoma tumor-initiating cells

**DOI:** 10.1007/s00401-014-1327-6

**Published:** 2014-08-06

**Authors:** Mark Barszczyk, Pawel Buczkowicz, Pedro Castelo-Branco, Stephen C. Mack, Vijay Ramaswamy, Joshua Mangerel, Sameer Agnihotri, Marc Remke, Brian Golbourn, Sanja Pajovic, Cynthia Elizabeth, Man Yu, Betty Luu, Andrew Morrison, Jennifer Adamski, Kathleen Nethery-Brokx, Xiao-Nan Li, Timothy Van Meter, Peter B. Dirks, James T. Rutka, Michael D. Taylor, Uri Tabori, Cynthia Hawkins

**Affiliations:** 1 The Arthur and Sonia Labatt Brain Tumor Research Centre, The Hospital for Sick Children, Toronto, ON Canada; 2 Department of Laboratory Medicine and Pathobiology, University of Toronto, Toronto, ON Canada; 3 Division of Pathology, The Hospital for Sick Children, Toronto, ON Canada; 4 Regenerative Medicine Program, Department of Medicine and Biomedical Sciences, Centre for Molecular and Structural Biomedicine, CBME/IBB, University of Algarve, Faro, Portugal; 5 Division of Hematology and Oncology, The Hospital for Sick Children, Toronto, ON Canada; 6 Brain Tumor Program, Texas Children’s Cancer Center, Houston, TX USA; 7 Division of Pediatric Hematology-Oncology, Virginia Commonwealth University, Richmond, VA USA; 8 Division of Surgery, The Hospital for Sick Children, Toronto, ON Canada

**Keywords:** Ependymoma, Telomerase, Telomerase inhibition, Imetelstat, TRAP

## Abstract

Pediatric ependymomas are highly recurrent tumors resistant to conventional chemotherapy. Telomerase, a ribonucleoprotein critical in permitting limitless replication, has been found to be critically important for the maintenance of tumor-initiating cells (TICs). These TICs are chemoresistant, repopulate the tumor from which they are identified, and are drivers of recurrence in numerous cancers. In this study, telomerase enzymatic activity was directly measured and inhibited to assess the therapeutic potential of targeting telomerase. Telomerase repeat amplification protocol (TRAP) (*n* = 36) and C-circle assay/telomere FISH/ATRX staining (*n* = 76) were performed on primary ependymomas to determine the prevalence and prognostic potential of telomerase activity or alternative lengthening of telomeres (ALT) as telomere maintenance mechanisms, respectively. Imetelstat, a phase 2 telomerase inhibitor, was used to elucidate the effect of telomerase inhibition on proliferation and tumorigenicity in established cell lines (BXD-1425EPN, R254), a primary TIC line (E520) and xenograft models of pediatric ependymoma. Over 60 % of pediatric ependymomas were found to rely on telomerase activity to maintain telomeres, while no ependymomas showed evidence of ALT. Children with telomerase-active tumors had reduced 5-year progression-free survival (29 ± 11 vs 64 ± 18 %; *p* = 0.03) and overall survival (58 ± 12 vs 83 ± 15 %; *p* = 0.05) rates compared to those with tumors lacking telomerase activity. Imetelstat inhibited proliferation and self-renewal by shortening telomeres and inducing senescence in vitro. In vivo, Imetelstat significantly reduced subcutaneous xenograft growth by 40 % (*p* = 0.03) and completely abolished the tumorigenicity of pediatric ependymoma TICs in an orthotopic xenograft model. Telomerase inhibition represents a promising therapeutic approach for telomerase-active pediatric ependymomas found to characterize high-risk ependymomas.

**Electronic supplementary material:**

The online version of this article (doi:10.1007/s00401-014-1327-6) contains supplementary material, which is available to authorized users.

## Introduction

Ependymomas represent the third most common central nervous system (CNS) tumor in children and mainly arise in young children under 5 years of age within the posterior fossa [34, 46]. Pediatric ependymomas are highly recurrent and chemoresistant entities that will often recur numerous times throughout a patient’s lifetime [32]. Current standard of care aims for complete surgical resection followed by radiotherapy. However, over 50 % of children with gross total resection will still experience tumor recurrence despite aggressive multimodal therapy [35]. Furthermore, radiation in young children is associated with long-term cognitive sequelae [17, 39]. The only widely accepted prognostic factor of outcome is extent of surgical resection, while histological grading has proven to be an unreliable and poor predictive factor [7, 38]. The lack of robust therapeutic and prognostic targets has contributed to poor 5-year progression-free survival (PFS) and overall survival (OS) rates of 23–45 and 50–64 %, respectively, and highlights the urgent need to identify targetable pathways in pediatric ependymoma to improve patient outcomes [17, 39].

Telomeres are regions of repetitive DNA found at the end of chromosomes that shorten during cell division due to incomplete DNA replication [9]. Following continued proliferation, telomeres erode to a critically short length and induce a growth-arrested state known as senescence [10]. However, stem cells and over 90 % of cancers express telomerase, which employs its RNA component (hTR) to bind to telomeric sequences and synthesize telomeric hexanucleotide repeats de novo using a reverse transcriptase domain (hTERT), preventing telomere erosion and permitting sustained proliferation [16, 43]. Telomerase has been found to be critically important for the maintenance of tumor-initiating cells (TICs), which are chemoresistant cells able to repopulate the tumor from which they are identified, and drivers of recurrence in numerous cancers [5, 20]. ALT represents a telomerase-independent mechanism of telomere maintenance that relies upon homologous recombination machinery to maintain telomeres [3]. Although this mechanism is relatively rare in most cancers, ALT appears in 30–50 % of pediatric and adult high-grade gliomas (HGGs) and its prevalence in other brain tumors such as pediatric ependymoma is yet to be elucidated [1].

Numerous studies have suggested telomerase contributes to recurrence in pediatric ependymoma by assessing hTERT expression, as it is believed to be the rate-limiting factor for telomerase activation. Immunohistochemical detection methods have demonstrated that high expression levels of the catalytic hTERT subunit predicts poor PFS and OS in primary ependymoma both alone and as a model of telomere dysfunction [23, 36]. Unfortunately, the antibody used in these studies has subsequently been found to cross-react with nucleolin and is thus not rigorous enough for routine clinical use [44]. Furthermore, hTERT mRNA expression was studied and found to be a strong predictor of OS in pediatric ependymoma [25]. However, studies have found up to a 30 % discordance between hTERT mRNA expression and telomerase activity with the latter a more sensitive test [19]. Most recently, hypermethylation of the hTERT promoter has been associated with increased hTERT mRNA expression and has been found to predict both PFS and OS in pediatric ependymoma [4]. However, the mechanism of hTERT upregulation following promoter hypermethylation remains unclear. Although all of these studies suggest telomerase represents a prognostic biomarker and a therapeutic target in pediatric ependymoma, robust detection of telomerase activity is required to definitely address the importance of telomerase in this tumor type.

In this study, we assessed telomerase activity directly using the telomerase repeat amplification protocol (TRAP) to undeniably determine the prevalence of telomerase as a telomere maintenance mechanism in pediatric ependymoma and to determine whether telomerase enzymatic activity can predict recurrence. Telomerase activity was then directly targeted using the telomerase inhibitor Imetelstat [20] in pediatric ependymoma cell models and patient-derived xenografts to determine the effect on ependymoma tumor initiating potential.

Our results show that pediatric ependymomas rely exclusively on telomerase as a mechanism of telomere maintenance and that telomerase activity is associated with increased recurrence rates and higher mortality. Furthermore, telomerase inhibition was able to reduce ependymoma growth in vitro and in vivo along with total inhibition of TIC tumorigenicity. These findings suggest that telomerase activity may comprise a promising prognostic biomarker and a therapeutic target in a tumor type that lacks effective prognostic and chemotherapeutic options.

## Materials and methods

### Patient samples and clinical data

Patient samples and clinical data used were gathered from primary and recurrent pediatric ependymomas operated on between 1990 and 2013 at The Hospital for Sick Children (Sick Kids, Toronto, ON, CA) following informed consent and approval by the institutional Research Ethics Board. 36 fresh-frozen samples were used for telomerase activity detection, 97 formalin-fixed, paraffin-embedded (FFPE) samples were used for C-circle analyses, telomere FISH and ATRX staining, 18 fresh-frozen samples were used for hTERT promoter mutational analysis, 24 fresh-frozen and FFPE samples were used for hTERT promoter hypermethylation analysis, 11 FFPE samples were used for *C11orf95*-*RELA* fusion subgrouping and 23 fresh-frozen and FFPE samples were used for CIMP subgrouping. Table 1 provides a clinical description of the patient cohort used for assessing the prognostic potential of telomerase activity, while Table S1 provides individual clinical data on all samples used in the study where available.

**Table 1:** Clinical characteristics and telomerase activity status of pediatric ependymoma cohort

Clinical characteristics	Patients		5-year PFS			5-year OS		
	#	%	%	SE	Log-rank (*p*)	%	SE	Log-rank (*p*)
Age >3 years								
Yes	25	69	51	12	0.36	46	15	0.36
No	11	31	18	16		36	21	
Sex								
Male	26	72	44	13	0.30	45	16	0.20
Female	10	28	35	16		42	17	
Tumor location								
Supratentorial	13	36	32	16	0.16	45	21	0.97
Infratentorial	23	64	46	13		42	16	
Grade								
2	14	39	42	17	0.45	69	19	0.02*
3	22	61	41	12		25	14	
Resection								
GTR	21	58	49	14	0.35	54	17	0.07
Subtotal	13	36	31	15		33	18	
Biopsy	2	6	50	35		50	35	
Radiation								
Yes	25	69	51	12	0.03*	53	16	0.03*
No	11	31	20	16		21	17	
Chemotherapy								
Yes	21	58	40	14	0.94	45	16	0.75
No	15	42	43	15		41	20	
Telomerase activity								
Yes	23	64	29	11	0.03*	58	12	0.05*
No	13	36	64	18		83	15	

*PFS* Progression-free survival, *OS* overall survival, *SE* standard error, *GTR* gross total resection

* Significance as determined by log-rank statistics at *p* ≤ 0.05

### Telomerase repeat amplification protocol (TRAP)

The telomerase activity status of patient samples was assessed using the TeloTAGGG Telomerase PCR ELISA kit (Roche, Sandhoferstrasse, MA, NE) using 1 μg of lysate per sample and appropriate controls as previously described [36]. Telomerase activity of cell samples was assessed using a modified version of the gel-based TRAPeze Telomerase Detection kit (Millipore, Temecula, CA, USA) utilizing a Cy5-labeled forward primer (Cy5-ATTCCGTCGAGCAGAGTT). In brief, untreated, mismatch and Imetelstat-treated cells were lysed using CHAPS lysis buffer. Cell extract (1.2 µg), negative control (lysis buffer) and positive control extract (provided in kit) were then added to the master mix to yield a total volume of 50 μL. PCR amplification consisted of incubation at 30 °C for 30 min, followed by 35 cycles of 94 °C for 20 s, 56 °C for 30 s and 72 °C for 30 s. Approximately 30 μL of each PCR reaction was loaded onto a 12.5 % non-denaturing acrylamide gel and run for 4 h at 250 V. Telomerase amplification products were imaged using the FluorChem^®^ Q MultiImage III system (ProteinSimple, Santa Clara, Ca, USA). The presence of a 6 base-pair banding ladder indicated active telomerase.

### Taqman genotyping assay

C228T and C250T hTERT promoter mutations were assessed in clinical samples and cell lines as previously described [28]. 25 ng of sample DNA was run per reaction in parallel with mutation-positive DNA serving as a positive control and sterile water serving as a negative control.

### Bisulfite conversion and sequenom mass spectrometry

To determine hTERT promoter hypermethylation and CIMP status using sequenom mass spectrometry, DNA isolated from either fresh-frozen or FFPE samples was bisulfite converted following kit instructions (Qiagen, EpiTect plus). hTERT promoter hypermethylation and CIMP status were then determined as previously described [4, 18].

### Fluorescence in situ hybridization (FISH)

ALT status was determined by telomere FISH using the Telomere PNA FISH Kit/Cy3 (Dako, Burlington, ON, CA) following a generalized protocol as previously described [37]. Telomere FISH was performed on 5-μm sections of pediatric ependymoma FFPE tissue microarrays containing tumor samples in triplicate alongside normal tissue controls and ALT-positive high-grade glioma as a positive control. Positivity was defined as showing very bright, intranuclear foci in at least 1 % out of the 200 total cells quantified per core, as well as having at least two cores scored. Scoring was performed on a Nikon Eclipse E400 fluorescent microscope (Nikon Instruments, Toronto, ON, CA) with appropriate filters at 1,000× magnification.

*C11orf95*-*RELA* fusion status was determined using ‘break-apart’ probes for the *RELA* gene as previously described [26]. FISH was performed on 5-μm sections of FFPE tissue. RP11-642F7 probe was labeled with spectrum green, while CH17-211O12 probe was labeled with spectrum orange. The BAC from the hydatidiform mole (CH17-211O12) was created at BACPAC Resources by Drs. Mikhail Nefedov and Pieter J. de Jong using a cell line created by Dr. Urvashi Surti. Fusion positivity was defined as more than 25 % of 200 quantified cells showing a ‘break-apart’ event. Scoring was performed on a Nikon Eclipse E400 fluorescent microscope (Nikon Instruments, Toronto, ON, CA) with appropriate filters at 1,000× magnification.

### Immunohistochemistry

To assess ALT status using ATRX expression, immunohistochemistry was performed as previously described [15]. 5-μm sections of pediatric ependymoma FFPE tissue microarrays containing tumor samples in triplicate alongside numerous control tissues were stained with rabbit anti-human ATRX antibody (HPA001906, Sigma-Aldrich) at a concentration of 1:600 overnight at 4 °C. The sections were scored for nuclear positivity based upon distribution (0, 0–25, >50) and intensity (light, strong). Samples were considered positive if two or more cores were scorable and if all scored cores showed more than 25 % of nuclei staining strongly, suggesting a lack of ATRX mutations and therefore lack of ALT. Cores were considered negative only if normal endothelial cells stained strongly, as these served as internal positive controls for each core. An ALT-positive high-grade glioma was stained in parallel as a positive control.

### C-circle assay

C-circle assay was used to determine the prevalence of ALT in patient samples as previously described [11]. Following DNA extraction from 51 FFPE samples using the RecoverAll™ Total Nucleic Acid Isolation Kit for FFPE (Life Technologies, Burlington, ON, CA), 16 ng of DNA was incubated in master mix containing 5 U of Φ29 polymerase for 8 h at 30 °C to allow for C-circle amplification. Quantitative polymerase chain reaction (qPCR) was then run on 2 ng of Φ29 polymerase amplified and non-amplified DNA in triplicate using a Lightcycler 480 (Roche). qPCR conditions used were 95 °C for 15 min followed by 35 cycles of 95 °C for 15 s and 54 °C for 2 min. The presence of C-circles was determined by calculating a ∆meanCp value between triplicate Φ29 polymerase amplified and non-amplified runs for each sample. Samples with a ∆meanCp value greater than +0.2 were considered C-circle positive while samples with a ∆meanCp value less than −0.2 were considered C-circle negative. ALT-positive fibroblasts (GMA47) were used as a positive control, while ALT-negative cervical cancer cells (HeLa) were used as a negative control.

### Cell lines

Established BXD-1425EPN (BXD) supratentorial pediatric ependymoma cells were acquired and grown as previously described [45]. R254 cells were derived from a supratentorial pediatric ependymoma and cultured as an established cell line in DMEM/F12 (Invitrogen, Burlington, ON, CA) supplemented with 15 % fetal bovine serum (Invitrogen) and 1× penicillin/streptomycin (Invitrogen). E520 TICs were derived from an infratentorial pediatric ependymoma and cultured as previously described [18].

All three cell lines were further characterized for hTERT promoter mutations, hTERT promoter hypermethylation and telomerase activity. Supratentorial lines (BXD, R254) were characterized for *C11orf95*-*RELA* fusion status and previously reported copy number alterations, while E520 infratentorial cells are known to be Group A/CIMP (+) [18].

### SNP array hybridization and data analysis

Genomic DNA from ependymoma cell lines R254 and BXD was hybridized to the CytoScan HD Array (Affymetrix, Santa Clara, CA, USA). DNA digestion, labeling and array hybridization was performed by The Centre for Applied Genomics (TCAG) at The Hospital for Sick Children (Toronto, ON, Canada). CEL files with raw chip intensity data were analyzed for copy number alterations at specific loci previously reported to be associated with ependymoma using segmentation algorithm in Partek Genomics Suite (v6.6). Copy number was inferred from differences between the two cell lines and HapMap baseline. Diploid copy number was assumed between 1.5 and 2.5 copies. All remaining parameters were used at default settings.

### *In vitro* telomerase inhibition

BXD (5 × 10^5^), R254 (1 × 10^5^) and E520 (1 × 10^5^) cells were seeded weekly in P100 plates (BD Biosciences, Mississauga, ON, CA) with fresh media containing either 5 µM of the telomerase inhibitor Imetelstat (Geron, Menlo Park, CA, USA) or scrambled mismatch oligonucleotide control (Geron) in parallel with an untreated control. Imetelstat consists of a palmitoylated 13-mer thiophosphoramidate oligonucleotide sequence (5′-Palm-TAGGGTTAGACAA-3′) with complementarity and high affinity to the hTR of telomerase that directly inhibits telomerase activity, while the mismatch control differs by four residues (5′-Palm-TAGGTGTAAGCAA-3′). Untreated, mismatch control and Imetelstat treatment groups had media containing the respective compounds replenished midweek. At the end of each week, cell number and viability were determined using the Vi Cell XR cell counter (Beckman Coulter, Mississauga, ON, CA), cell pellets were collected for subsequent analysis and the appropriate number of cells for each cell line was replated for further treatment until growth arrest was observed. Population doublings were assessed using the formula: (number of cells collected/number of cells seeded)/log 2. All experiments were performed in triplicate.

For MST-312 (Sigma-Aldrich, Oakville, ON, CA) telomerase inhibition, 1 × 10^5^ cells were seeded in 6-well plates (BD Biosciences, Mississauga, ON, CA) and left overnight to attach in triplicate for each dose. MST-312 was administered in varying doses (0–4 µM) for 72 h and cell number and viability were determined using the Vi Cell XR cell counter (Beckman Coulter, Mississauga, ON, CA). Cell pellets were also collected at each dose for telomerase activity assessment. Effective doses were then chosen for BXD (2 µM), R254 (2 µM) and E520 (4 µM) cells for subsequent senescence, γH2AX and cell cycle analysis.

### Telomere restriction fragment (TRF) assay

Telomere length was determined using the TeloTAGGG Telomere Length Assay Kit (Roche) according to manufacturer’s instructions. 1.5 µg of DNA was used per sample and average telomere length was calculated by dividing each lane into 20 equally sized rectangles, quantifying density with ImageJ software (http://rsb.info.nih.gov/ij/) and using the formula length = Σ(density)/Σ(density/distance on gel). Appropriate positive and negative controls provided with the kit were included with each run.

### Immunofluorescence

Immunofluorescence was performed as previously described [41]. Primary antibody used was γH2AX (1:1,000, Millipore). Slides were viewed and images captured using an Eclipse E400 fluorescent microscope equipped with a DXM1200F camera (Nikon, Melville, NY, USA). Image analyses were performed using ImageJ software (http://rsb.info.nih.gov/ij/). For γH2AX foci quantification, 50 cells in random fields of view were scored in triplicate for untreated, mismatch control and Imetelstat-treated cells.

### $\beta$-Galactosidase assay

Senescence was determined using a β-galactosidase Staining kit (Cell Signalling Technology, Beverly, MA, USA). 1 × 10^5^ untreated, mismatch control or Imetelstat-treated cells were seeded on glass coverslips and left overnight to attach in triplicate. Following kit instructions, images were captured using an Eclipse E400 microscope (Nikon) and 50 cells in random fields of view were quantified for blue coloration indicating senescence.

### Clonogenic assay

R254 cells that were either untreated, or treated with mismatch or Imetelstat, were seeded in P100 plates (BD Biosciences) in triplicate and cultured for 2 weeks. Media was removed and cells were fixed and stained with crystal violet solution containing 2.5 mg/ml crystal violet (Sigma-Aldrich, Oakville, ON, CA), 80 % methanol (Sigma-Aldrich) and 3.5 % formaldehyde (Sigma-Aldrich). Crystal violet solution was then washed off and colonies were manually quantified.

### Flow cytometry

Approximately 5 × 10^5^ to 1 × 10^6^ cells were prepared for cell cycle arrest analysis as previously described [41]. A Becton–Dickinson LSRII 15-color analyzer (Mississauga, ON, CA) was used to detect 1 × 10^4^ events in triplicate for untreated and MST-312 treated cells. Collected data were then analyzed using FlowJo flow cytometry analysis software (http://www.flowjo.com/).

### Sphere-forming assay

Sphere-forming analyses were performed as previously described [33]. In brief, E520 neurospheres were dissociated and plated in quadruplicate in a 96-well plate (BD Biosciences) in 100 μL of stem cell media in triplicate. Cell dilutions ranged from 200 cells/well to 4 cells/well. Spheres were allowed to form for 14 days at 37 °C and then sphere number was quantified for each well and plotted against the number of cells seeded per well. In addition, the percentage of wells not containing spheres was calculated and plotted against the number of seeded cells per well. Regression lines were plotted and the *x*-intercept was determined, representing the number of cells required to form one tumor sphere in every well.

### Orthotopic telomerase inhibition

5.0 × 10^4^ E520 cells transfected with luciferase using lentivirus were suspended in 3 μL of stem cell media and injected into the cerebral hemisphere (1 mm to the right of the midline, 1.5 mm anterior to the lambdoid suture and 3 mm deep) of 8- to 12-week-old male and female NSG mice as previously described [45]. Following 7 days, mice were injected subcutaneously with D-luciferin (Goldbio, St. Louis, MO, USA) at a concentration of 0.15 mg/mg to allow imaging using an IVIS Imaging System (PerkinElmer, Woodbridge, ON, CA). Mice with detectable tumors were randomly assigned into either PBS (*n* = 9) or Imetelstat (*n* = 8) treatment groups and injected intraperitoneally thrice weekly with Imetelstat (30 mg/kg) or PBS equivalent. Upon killing, tumors were both fixed in formalin and snap-frozen for subsequent analysis. Animal procedures were approved by the Sick Kids Animal Care Committee and performed in a facility approved by the Canadian Council of Animal Care.

### Subcutaneous telomerase inhibition

5.0 × 10^4^ E520 TICs suspended in 200 µl of 1:1 Matrigel (BD Biosciences)/PBS (Invitrogen) solution were injected into the flank of NOD/SCID/Gamma (NSG) immunodeficient mice. Following 9 days, tumor presence was validated by palpation and mice possessing tumors were randomly assigned into PBS or Imetelstat treatment groups (*n* = 6/group). Mice were then injected intraperitoneally three times weekly with Imetelstat (30 mg/kg) or PBS equivalent. Tumor volume was quantified weekly using digital calipers and all mice were killed following 5 weeks of treatment. Tumors were both fixed and snap-frozen for subsequent analysis. Animal procedures were approved by Sick Kids Animal Care Committee and performed in a facility approved by the Canadian Council of Animal Care.

### Tumorigenicity assay

3.0 × 10^4^ E520 untreated or Imetelstat-pretreated TICs (34 weeks) were suspended in 2 μl of stem cell media and injected into the cortex (1 mm to the right of the midline, 1.5 mm anterior to the lambdoid suture and 3 mm deep) of NSG immunodeficient mice as previously described [45]. Mice were monitored daily until signs of morbidity were observed and were killed for subsequent histopathological analysis. Mice not displaying signs of morbidity were killed following 90 days post-injection. Brains from mice were removed, fixed in formalin and assessed by a neuropathologist (CH) for tumor growth. Two mice were lost to follow-up. Animal procedures were approved by Sick Kids Animal Care Committee and performed in a facility approved by the Canadian Council of Animal Care.

### Statistical analysis

Statistical analyses were performed using SPSS v21 (IBM Corp, Armonk, NY, USA). Kaplan–Meier methods were used to determine survival statistics on patient progression and survival based on age (>3 years), sex, tumor location, grade, level of resection, radiation, chemotherapy and telomerase status, as well as mouse survival following orthotopic injection. Log-rank tests were performed to determine univariate significance (*p* ≤ 0.05) of Kaplan–Meier survival curves. Unpaired, two-tailed Student’s *t* test was used to determine statistical significance (*p* ≤ 0.05) of cell counts, viability, immunofluorescent positivity, colony and sphere formation, senescence, tumor growth and telomere length. Chi squared tests were performed when testing for significant (*p* ≤ 0.05) associations between biological features.

## Results

### Telomerase is the sole telomere maintenance mechanism in pediatric ependymoma and predicts recurrence and survival

To determine the prevalence of telomerase activity in pediatric ependymoma, TRAP assays were performed on 36 fresh-frozen primary ependymoma cases. 64 % (23/36) of ependymomas were found to possess active telomerase (Table 1). Since recently identified mutations and hypermethylation within the hTERT promoter have been suggested to drive telomerase activation, the association of telomerase activity with either of these mechanisms was investigated [4, 12, 13]. While none (0/18) of the pediatric ependymomas screened for C228T and C250T hTERT promoter mutations using a Taqman assay were found to harbor mutations, 67 % (16/24) of ependymomas were hypermethylated at the hTERT promoter upon sequenom analysis (Table S1). Hypermethylation was not significantly (*p* = 0.67) associated with telomerase activity within our limited cohort.

The association of telomerase activity with recently identified subgrouping of infratentorial (CpG island methylator phenotype (CIMP)) and supratentorial (*C11orf95*-*RELA* fusion) pediatric ependymomas was also investigated [18, 26, 27, 40]. 78 % (18/23) of infratentorial ependymomas were determined to be Group A/CIMP (+) upon sequenom analysis (Table S1), while 45 % (5/11) of supratentorial cases harbored *C11orf95*-*RELA* fusions upon interphase FISH (Fig. S1; Table S1). Neither CIMP (*p* = 0.64) nor *C11orf95*-*RELA* fusion status (*p* = 1.00) was significantly associated with telomerase activity suggesting that telomere maintenance is independent of subgroup status.

**Fig. 1:**
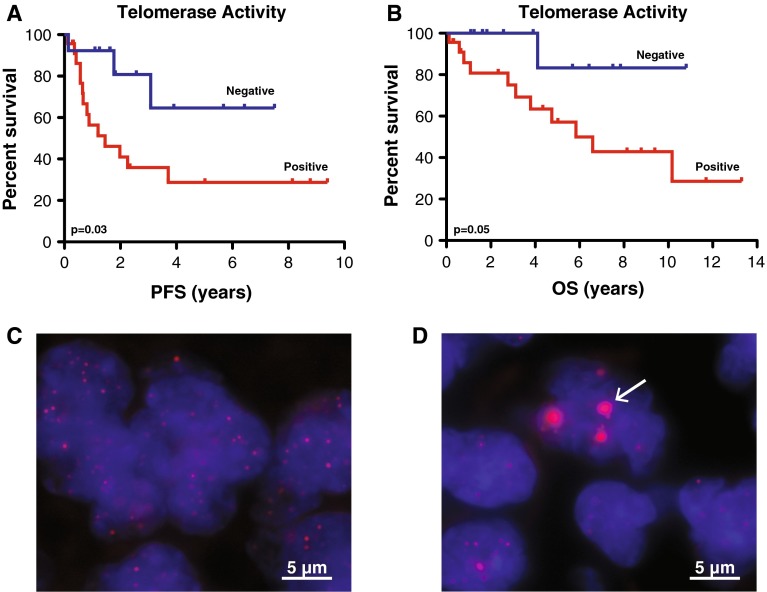
Telomerase activity predicts progression and survival in pediatric ependymoma, while no cases rely on ALT to maintain telomeres. Kaplan–Meier analysis (*n* = 36) showed children with ependymomas possessing active telomerase (positive) had reduced progression-free survival (**a**) and overall survival (**b**) compared to children whose tumors lacked active telomerase (negative). Telomere FISH showed a lack of ultrabright intranuclear foci (**c**) in 56 primary ependymomas indicating a lack of ALT, while these foci (*arrow*) could be observed in an ALT-positive high-grade glioma positive control (**d**). Significance was determined using log-rank statistics and images were taken at 1,000× magnification. *PFS* progression-free survival, *OS* overall survival

Kaplan–Meier estimates and log-rank survival analyses were performed to determine whether children whose tumors possessed telomerase activity were more likely to experience progression or mortality. Children harboring telomerase-active tumors showed reduced 5-year PFS (29 ± 11 vs 64 ± 18 %; *p* = 0.03) and OS (29 ± 13 vs 83 ± 15 %; *p* = 0.05) rates compared to children whose tumors lacked telomerase activity (Fig. 1a, b; Table 1). Assessment of telomerase activity separately within either the supratentorial or infratentorial compartment showed activity predicted reduced 5-year PFS within the infratentorial region (31 ± 14 vs 64 ± 21 %; *p* = 0.05). Upon multivariate survival analysis, TRAP activity was associated with the greatest hazard ratio and approached significance most closely (HR = 5.67, *p* = 0.10); however, low cohort size limited statistical power (Table S2).

Telomere FISH (*n* = 56), C-circle analysis (*n* = 51) and ATRX immunohistochemistry (*n* = 41) were then performed on a combined total of 76 unique primary pediatric ependymomas to determine whether ependymomas also rely on ALT as a mechanism of telomere maintenance. 75 % (57/76) of cases were assessed for ALT using more than one technique. Interestingly, none of the primary pediatric ependymomas showed evidence of ALT upon telomere FISH (Fig. 1c, d; Table S1), C-circle analysis (Table S1), or ATRX staining (Fig. S2; Table S1). In addition, although previous work has shown that 100 % of recurrent ependymomas (8/8) possessed active telomerase, the prevalence of ALT in recurrent ependymoma remained to be elucidated [29]. Using telomere FISH (*n* = 16) and ATRX staining (*n* = 12), with 33 % (7/21) of cases being assessed with both techniques, we found that recurrent ependymomas did not rely on ALT as a mechanism of telomere maintenance (Table S1). Since telomerase activity comprises a hallmark event observed in the majority of pediatric ependymomas and specifically identifies high-risk patients with decreased PFS and OS, telomerase inhibition was investigated as a novel therapeutic strategy.

### Telomerase inhibition attenuates proliferation in vitro by shortening telomeres and inducing senescence

**Fig. 2:**
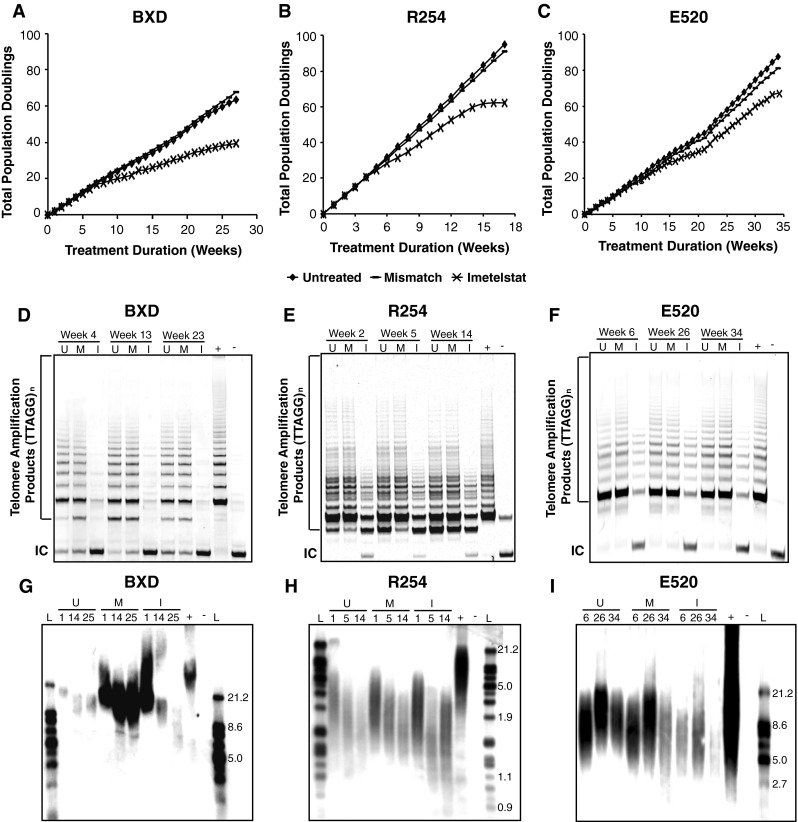
Imetelstat reduced proliferation, inhibited telomerase and shortened telomeres in three pediatric ependymoma cell lines. Prolonged Imetelstat treatment of *BXD* (**a**), *R254* (**b**) and *E520* (**c**) cells inhibited proliferation following 8, 6 and 16 weeks, respectively. TRAP assay showed that *BXD* (**d**), *R254* (**e**) and *E520* (**f**) cells treated with Imetelstat had a marked reduction in telomerase activity throughout treatment compared to untreated and mismatch control cells as indicated by a reduced banding pattern. TRF assay also showed *BXD* (**g**), *R254* (**h**) and *E520* (**i**) cells treated with Imetelstat underwent a progressive decrease in telomere length as determined by lower banding compared to untreated and mismatch control cells as treatment duration increased (weeks). Positive control (+) for TRAP and TRF assays were kit provided telomerase-positive lysate and control DNA, respectively. Negative control (−) for TRAP and TRF assays were lysis buffer and sterile water, respectively. TRF ladder represents kbps. *U* untreated, *M* mismatch, *I* Imetelstat, *IC PCR* internal control, *L* ladder

We investigated the effect of telomerase inhibition on proliferation using two established pediatric ependymoma cell lines (BXD, R254) and a primary TIC line (E520). Characterization of these cell models for hTERT promoter mutations and hypermethylation, telomerase activity, subgroup affiliation and previously reported copy number alterations indicated these cell models share characteristic features common to pediatric ependymoma (Table S3) [14, 23]. BXD, R254 and E520 cells treated with the telomerase inhibitor Imetelstat showed decreased proliferation following prolonged treatment compared to untreated and scrambled oligonucleotide mismatch control cells (Fig. 2a–c; *p* < 0.05). All three in vitro models treated with Imetelstat displayed significant reduction of telomerase activity throughout treatment (Fig. 2d–f), as well as progressive telomere shortening compared to control cells (Fig. 2g–i). Onset of growth arrest was not associated with initial telomere length and a lack of ALT-associated banding patterns comprised of long and heterogeneous telomere length upon TRF (Fig. 2g–i) indicated that cells did not convert to an ALT phenotype as an escape mechanism despite prolonged treatment duration.

**Fig. 3:**
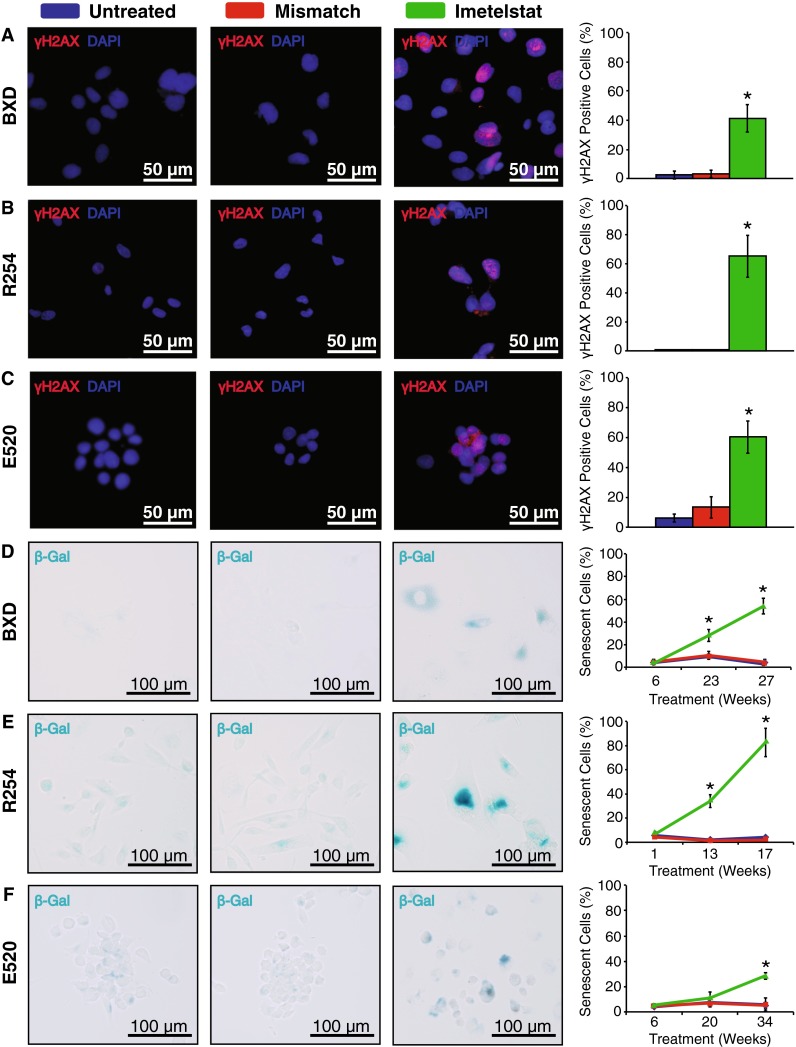
Imetelstat-treated cells displayed an activated DNA damage response associated with a progressive increase in senescence. Immunofluorescence showed *BXD* (**a**), *R254* (**b**) and *E520* (**c**) cells had increased γH2AX staining compared to untreated and mismatch control cells following 27, 17 and 34 weeks of treatment, respectively. β-galactosidase (β-gal) staining showed a progressive increase in cells undergoing senescence in *BXD* (**d**), *R254* (**e**) and *E520* (**f**) cells. All images were taken at 200× magnification and *scale bars* represent either 50 or 100 μm as indicated. *Error bars* represent ± SD of the mean. *Asterick* represents significance at *p* < 0.05 compared to both untreated and mismatch control

Once telomeres erode to a critically short length, they become dysfunctional and activate a DNA damage response mediated by γH2AX, which ultimately results in senescence [6]. All three cell models treated with Imetelstat showed evidence of γH2AX foci in ~40–60 % of cells by the end of treatment, while almost none of the untreated or mismatch control cells showed evidence of DNA damage (Fig. 3a–c; *p* < 0.05). BXD, R254 and E520 cells showed a progressive increase in the proportion of Imetelstat-treated cells undergoing senescence, whereby 60, 80 and 30 % of cells, respectively, underwent senescence by the end of treatment (Fig. 3d–f; *p* < 0.05). Although R254 cells showed a 15 % (*p* < 0.05) increase in apoptosis in the last 3 weeks of treatment, BXD and E520 cells did not undergo any significant cell death (data not shown).

Previous studies have shown Imetelstat may exert off-target cytoskeletal effects characterized by cell rounding morphological changes, loss of adherence and reduced proliferation [22]. To determine whether similar off-target effects contributed to the observed results, cell surface area was quantified at the end of Imetelstat treatment for each cell line (Fig. S3). Cell surface area was either unchanged or increased in all three cell models, suggesting that the previously reported off-target cytoskeletal effects did not influence the observed results.

To validate telomerase as an effective therapeutic target in pediatric ependymoma, cells were treated with a second telomerase inhibitor, MST-312. Although low-dose, long-term (>28 day) MST-312 treatment has been shown to reduce proliferation and shorten telomeres, high-dose, short-term (3 day) MST-312 treatment reduces proliferation and induces DNA damage localized to telomeres [31, 41]. Following 72-h treatment of BXD, R254 and E520 ependymoma cells with MST-312, all three cell lines showed a dose-dependent decrease in proliferation (Fig. S4a–c; *p* < 0.05) and telomerase activity (Fig. S4d–f). Growth arrest was not associated with a reduction in viability or increase in senescence (data not shown). However, an increase in γH2AX-positive nuclei was observed in all three cell lines (Fig. S4g–i), with two of the three cell models (BXD, R254) displaying an accumulation of cells within the G2 cell phase, suggestive of G2/M cell cycle arrest (Fig. S4j–l). Imetelstat was subsequently chosen to study telomerase inhibition in vivo due to its defined mechanism and progress in clinical trials.

### Telomerase inhibition shortens telomeres and reduces tumor growth in vivo

To study the effect of telomerase inhibition in vivo, E520 TICs were used to generate an orthotopic model of pediatric ependymoma. Established tumors treated with Imetelstat did not result in improved overall survival (Fig. S5a; *p* = 0.59) or reduced tumor growth (Fig. S5b, c; *p* > 0.05) compared to control mice treated with PBS. Since Imetelstat-treated tumors failed to show significant signs of telomerase inhibition compared to PBS control mice (Fig. S5d), we concluded that either Imetelstat was not crossing the blood–brain barrier (BBB) effectively in our model or the E520 cells grew too quickly resulting in death of the animals before telomere attrition could occur sufficiently for a therapeutic effect. Thus, as an alternative, telomerase inhibition was assessed in a subcutaneous model to circumvent BBB penetration issues and permit longer treatment duration in this aggressive tumor model.

**Fig. 4:**
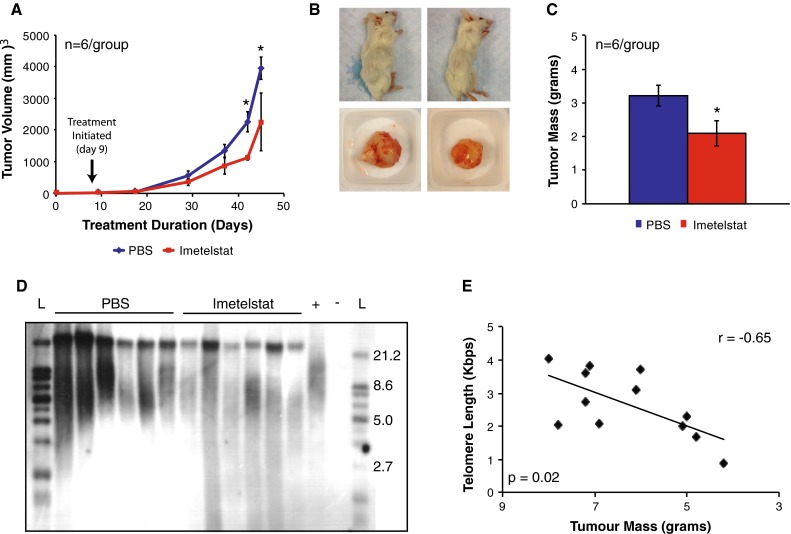
Imetelstat reduced E520 subcutaneous ependymoma growth and shortened telomeres (**a**). Following 5 weeks of treatment, Imetelstat-treated mice possessed tumors with average volumes 40 % smaller than PBS-treated mice (**b**). Upon killing (5 weeks post-treatment), Imetelstat-treated tumors appeared smaller than PBS-treated tumors and weighed 35 % less (**c**). **d** TRF showed Imetelstat-treated tumors had significantly shorter telomeres than PBS-treated mice, as determined by lower banding. **e** A significant (*p* = 0.02) Pearson product-moment correlation (*r* = −0.65) existed between telomere length and tumor mass. (*n* = 6 mice/group). *Error bars* represent ± SEM of the average for **a** and ± SD for **c**. Positive control (+) for TRF assay was kit provided DNA, while negative control (−) was sterile water. Ladder (L) is in kbps. *Asterick* represents significance at *p* < 0.05 compared to PBS control

Imetelstat-treated mice were found to possess subcutaneous tumors 40 % smaller than vehicle-treated mice following 5 weeks of treatment (Fig. 4a, b; *p* = 0.03). Imetelstat-treated tumors also weighed 35 % less than vehicle-treated mice (Fig. 4c; *p* = 0.04). Mice treated with the telomerase inhibitor Imetelstat were found to have significantly (*p* < 0.01) shorter average telomere lengths compared to PBS control mice (5.3 ± 0.96 vs 7.6 ± 0.66 kbps) (Fig. 4d). In addition, a lack of long and heterogeneous telomere length upon TRF once again indicated that despite prolonged treatment, tumors did not convert to an ALT phenotype (Fig. 4d). There was a significant correlation (*r* = −0.65, *p* = 0.02) between telomere length and tumor mass, whereby tumors with the smallest mass also possessed the shortest telomeres (Fig. 4e). These results highlight that telomerase inhibition can reduce pediatric ependymoma growth in vivo.

### Telomerase inhibition reduces self-renewal and abolishes tumorigenicity in pediatric ependymoma

**Fig. 5:**
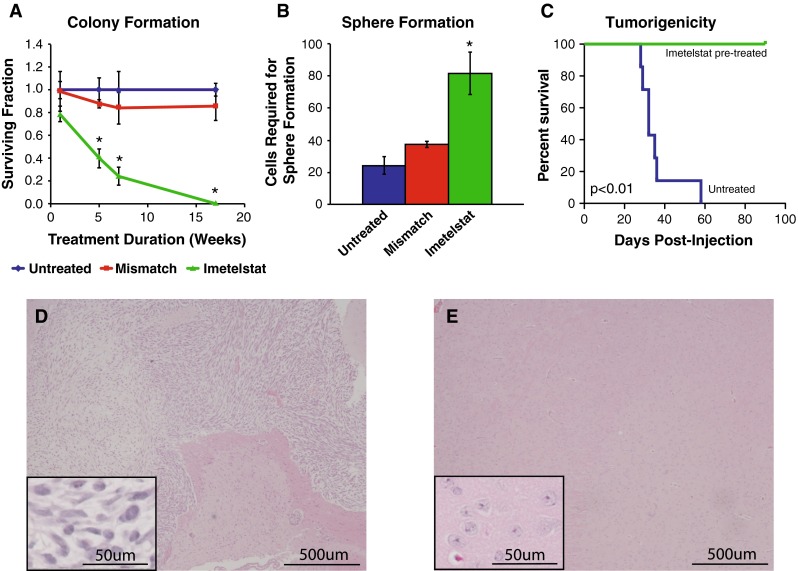
Imetelstat reduced self-renewal and tumorigenicity of pediatric ependymoma cells. **a** Colony forming assay showed Imetelstat progressively inhibited the self-renewal of R254 ependymoma cells, with complete inhibition by week 17 of treatment. **b** Sphere-forming assay showed a 75 % increase of cells required to be seeded to generate at least one sphere in each of four wells following 34 weeks of treatment. **c** Kaplan–Meier survival analysis showed mice injected supratentorially with Imetelstat-pretreated E520 cells (34 weeks) were asymptomatic at 90 days while mice injected with untreated E520 cells all required killing by day 60 (*n* = 7/group). Upon pathological analysis, all mice injected with untreated cells possessed tumors (**d**), while no mice injected with Imetelstat-pretreated cells showed evidence of neoplastic growth (**e**). Images were captured at 40× magnification with 200× inlets shown in *bottom left corners*. Student’s *t* test was used to determine significance in **a** and **b** while log-rank statistics were used to test significance in **c**. *Error bars* represent ± SD of triplicates

Since pediatric ependymomas are highly recurrent, we thought it was critical to determine whether telomerase inhibition attenuates the self-renewal and tumorigenic capacity of cells that may contribute to recurrence. Imetelstat induced a progressive decrease in the self-renewal of R254 cells throughout treatment, achieving complete inhibition of self-renewal following 17 weeks (Fig. 5a; *p* < 0.05). Similarly, using a sphere-forming assay to survey the self-renewal of E520 TICs, Imetelstat inhibited self-renewal by 75 % compared to untreated or mismatch control cells following 34 weeks of treatment (Fig. 5b; *p* < 0.01). Finally, untreated and Imetelstat-pretreated E520 cells (34 weeks) were injected intracranially into mice to assess whether telomerase inhibition attenuates tumorigenicity in vivo. Following 90 days, none of the mice injected with Imetelstat-pretreated cells showed any clinical evidence of tumor formation, while all of the mice injected with untreated E520 cells required killing (Fig. 5c). Histopathological examination revealed tumor formation in all mice that received E520 controls, while none of the mice injected with pretreated cells showed evidence of tumor formation (Fig. 5d, e). Therefore, these observations suggest loss of self-renewal and tumor initiating capacity of ependymoma cells in vitro and in vivo following telomerase inhibition.

## Discussion

Our work has shown that pediatric ependymomas rely exclusively on telomerase activity as a mechanism of telomere maintenance. Furthermore, telomerase activity is associated with increased progression and when inhibited, results in the loss of proliferation, self-renewal and tumorigenicity. These findings provide novel insight into the importance of telomerase as a predictor of outcome and as a therapeutic target in pediatric ependymoma.

This study is unique because we have utilized TRAP, the gold standard of telomerase enzymatic activity detection, as a method of studying telomerase activity in pediatric ependymoma. TRAP is superior to previously utilized methods of telomerase detection in pediatric ependymoma as it specifically detects telomerase activity in ependymomas. There are currently no specific antibodies for telomerase available [44] and hTERT mRNA analysis has shown poor correlation between TERT mRNA expression levels and enzymatic activity, albeit this discordance has not been shown specifically in pediatric ependymoma [19]. Our work has also shown that hTERT promoter mutations and methylation are not good surrogates for telomerase activity in pediatric ependymoma. The TRAP assay is a simple and robust method, utilizing only 1 μg of protein (or ~2 mm^3^ piece of tissue) for activity detection and could be introduced into clinical practice as a method to undeniably assess functional telomerase.

By comprehensively investigating telomere maintenance mechanisms in pediatric ependymoma, we have found that these tumors rely on telomerase to maintain telomeres or do not maintain telomeres at all, as none of the tumors screened for ALT were positive. Furthermore, to recur, pediatric ependymomas are highly reliant on telomerase (Fig. 1a). A previous study highlights the importance of telomerase for recurrence, whereby all recurrent ependymomas tested (8/8) had detectable telomerase activity [29], while none of the recurrent ependymomas screened (0/21) in our study showed evidence of ALT. Therefore, pediatric ependymomas lacking telomerase activity are unable to maintain telomeres and proliferative indefinitely, suggesting that less aggressive therapeutic intervention may be offered for children with telomerase-negative tumors. In fact, previous work has shown that pediatric gliomas lacking telomere maintenance undergo telomere shortening upon recurrence, and where primary tumors possessed short telomeres, spontaneous growth arrest was observed [37]. Telomerase activity was also not associated with CIMP or *C11orf95*-*RELA* fusion status, suggesting telomerase inhibition may be a useful therapeutic strategy with broad applicability. Our results show that in pediatric ependymoma, telomerase is an abundant target with few escape mechanisms thus representing an ideal therapeutic target that may aid in preventing recurrence.

Although numerous studies have shown reduced pediatric ependymoma growth in vitro, our study is one of the first to show significant growth inhibition in vivo [8, 18, 21, 24, 30, 42]. Telomerase inhibition reduced proliferation in two established cell lines and a primary TIC line (Fig. 2a–c) through shortening of telomeres (Fig. 2g–i) and induction of senescence (Fig. 3d–f). Imetelstat was also shown to reduce established tumor growth by 35–40 % with a correlation between shortened telomeres and tumor mass (Fig. 4). In both in vitro and in vivo models, despite prolonged telomerase inhibition, there was also no evidence that ependymoma cells switched to ALT to maintain their telomeres. Telomerase inhibition also appeared less efficacious toward reducing proliferation and inducing senescence of Group A/CIMP (+) cells compared to supratentorial cells (Figs. 2, 3), suggesting a subgroup-dependent efficacy of telomerase inhibition, however these observations could be attributable to differences in culture conditions and require further investigation to provide any sound conclusions. Telomerase inhibition significantly reduced self-renewal of both supratentorial and infratentorial cell models (Fig. 5a, b) and completely inhibited the tumorigenicity of CIMP-positive infratentorial ependymoma TICs in an orthotopic xenograft model (Fig. 5c–e). These results corroborate previous studies showing that Imetelstat can target cells with robust self-renewal and tumor initiating capacity in numerous cancer types including brain cancer, and suggest that these tumorigenic cell populations are highly dependent upon telomerase for continued proliferation [2, 5, 20]. Since telomerase inhibition requires a prolonged treatment duration to senesce cells and appears much more effective at attenuating self-renewal and tumorigenicity than proliferation, telomerase inhibition represents an ideal therapeutic approach following surgery when there is minimal residual disease, and should be taken into consideration in future clinical trials.

Despite these promising results, there are a number of limitations to this study. First and foremost, the requirement of fresh-frozen tissue to detect telomerase activity has limited our cohort size. In addition, although it has previously been shown that Imetelstat can cross the BBB in mice, Imetelstat was unable to effectively inhibit established orthotopic tumor growth, suggesting either poor BBB penetration in our model and/or rapid growth of our aggressive tumor model that prevented sufficient treatment duration [20]. Future clinical trials must take into consideration the BBB penetration of Imetelstat; however, our data provides important proof-of-principle evidence that telomerase inhibition represents a promising therapeutic strategy to pediatric ependymoma. Future work involves validating the prognostic potential of telomerase in a larger and prospective cohort, as well as utilizing telomerase inhibition as a combinational therapy and improving drug delivery to the brain.

In summary, telomerase is critical for the maintenance of telomeres in pediatric ependymoma and for sustaining cells with limitless proliferation that may contribute to recurrence. Telomerase inhibition represents a potentially promising maintenance therapy for telomerase-positive pediatric ependymomas following surgical resection when there is minimal residual disease.

## Electronic supplementary material

Below is the link to the electronic supplementary material.

**Fig. S1** ‘Break-apart’ *RELA* FISH probes identify translocation positive and negative supratentorial ependymoma cases. (a) Co-localization of red and green FISH probes spanning different ends of *RELA* indicate this gene has not been involved in a translocation event characteristic of *C11orf95*-*RELA* fusion. (b) Separation of one set of *RELA* probes indicates a translocation event has occurred involving the *RELA* gene that is highly suggestive of a fusion with *C11orf95*. Images were captured at 1000X magnification.

**Fig. S2** Pediatric ependymomas do not rely on ALT as determined by ATRX staining. (a) Strong nuclear ATRX staining in 41 primary ependymomas suggests a lack of ALT phenotype. (b) An ALT-positive high-grade glioma positive control shows a lack of ATRX staining in tumor cells. Images were captured at 200X magnification.

**Fig. S3** Imetelstat does not exert off-target cytoskeletal effects upon pediatric ependymoma cells. Phase contrast microscopy shows surface area is constant in BXD cells (a), increased in R254 cells (b) and remains constant in E520 cells (c) following 27, 17 and 34 weeks of Imetelstat treatment, respectively. All images were taken at 200X magnification and error bars represent ± SD of the mean of surface area of triplicate cell counts of 50 cells. * represents significance at p < 0.05 compared to both untreated and mismatch control while n.s. indicates no significant change.

**Fig. S4** Telomerase inhibitor MST-312 reduced proliferation due to G2/M cell cycle arrest in two of three ependymoma cell models. 72-hour MST-312 treatment of BXD, R254 and E520 cells significantly reduced proliferation (a-c) and telomerase activity (d-f) in a dose-dependent manner. Using a dose of 2 μM for BXD and R254 cells and 4 μM for E520 cells, 72-hour treatment induced an increase of γH2AX-positive nuclei in all three cell lines (g-i) and decreased G1 cell populations while increasing G2 cell populations in BXD and R254 cells. Blue bars represent untreated cells while red bars represent treated cells. Error bars represent ± SD of triplicates. * represents significance at p < 0.05 using t test, while n.s. indicates no significant change. Positive control (+) for TRAP assay was kit provided telomerase-positive lysate, while negative control (-) was lysis buffer.

**Fig. S5** Imetelstat failed to improve survival, reduce growth and inhibit telomerase activity in an E520 established orthotopic ependymoma model. (a) Imetelstat-treated mice did not display improved survival compared to PBS controls. (b, c) Monitoring mice for the first 21 days of treatment did not show any significant reduction in tumor growth as determined by bioluminescent imaging. (d) TRAP assay showed Imetelstat-treated tumors possessed telomerase activity similar to PBS controls. Log-rank statistics were used to determine significance in (a) while student’s *t* test was used in (b) at p < 0.05. Error bars (b) represent ± SEM. Positive control (+) for TRAP assay was kit provided telomerase-positive lysate, while negative control (-) was lysis buffer. n.s. = not statistically significant.

Supplementary material 1 (EPS 67.5 mb)

Supplementary material 2 (EPS 128 mb)

Supplementary material 3 (EPS 23.3 mb)

Supplementary material 4 (EPS 15.5 mb)

Supplementary material 5 (EPS 24.2 mb)

Supplementary material 6 (XLSX 56 kb)

Supplementary material 7 (DOCX 36 kb)

Supplementary material 8 (DOCX 52 kb)

## References

[1] Abedalthagafi M, Phillips JJ, Kim GE, Mueller S, Haas-Kogen DA, Marshall RE, Croul SE, Santi MR, Cheng J, Zhou S, Sullivan LM, Martinez-Lage M, Judkins AR, Perry A (2013). The alternative lengthening of telomere phenotype is significantly associated with loss of ATRX expression in high-grade pediatric and adult astrocytomas: a multi-institutional study of 214 astrocytomas. Mod Pathol.

[2] Brennan SK, Wang Q, Tressler R, Harley C, Go N, Bassett E, Huff CA, Jones RJ, Matsui W (2010). Telomerase inhibition targets clonogenic multiple myeloma cells through telomere length-dependent and independent mechanisms. PLoS One.

[3] Bryan TM, Englezou A, Gupta J, Bacchetti S, Reddel RR (1995). Telomere elongation in immortal human cells without detectable telomerase activity. EMBO J.

[4] Castelo-Branco P, Choufani S, Mack S, Gallagher D, Zhang C, Lipman T, Zhukova N, Walker EJ, Martin D, Merino D, Wasserman JD, Elizabeth C, Alon N, Zhang L, Hovestadt V, Kool M, Jones DT, Zadeh G, Croul S, Hawkins C, Hitzler J, Wang JC, Baruchel S, Dirks PB, Malkin D, Pfister S, Taylor MD, Weksberg R, Tabori U (2013). Methylation of the TERT promoter and risk stratification of childhood brain tumours: an integrative genomic and molecular study. Lancet Oncol.

[5] Castelo-Branco P, Zhang C, Lipman T, Fujitani M, Hansford L, Clarke I, Harley CB, Tressler R, Malkin D, Walker E, Kaplan DR, Dirks P, Tabori U (2011). Neural tumor-initiating cells have distinct telomere maintenance and can be safely targeted for telomerase inhibition. Clin Cancer Res.

[6] d’Adda di Fagagna F, Reaper PM, Clay-Farrace L, Fiegler H, Carr P, Von Zglinicki T, Saretzki G, Carter NP, Jackson SP (2003). A DNA damage checkpoint response in telomere-initiated senescence. Nature.

[7] Ellison DW, Kocak M, Figarella-Branger D, Felice G, Catherine G, Pietsch T, Frappaz D, Massimino M, Grill J, Boyett JM, Grundy RG (2011). Histopathological grading of pediatric ependymoma: reproducibility and clinical relevance in European trial cohorts. J Negat Results Biomed.

[8] Gilbertson RJ, Bentley L, Hernan R, Junttila TT, Frank AJ, Haapasalo H, Connelly M, Wetmore C, Curran T, Elenius K, Ellison DW (2002). ERBB receptor signaling promotes ependymoma cell proliferation and represents a potential novel therapeutic target for this disease. Clin Cancer Res.

[9] Harley CB, Futcher AB, Greider CW (1990). Telomeres shorten during ageing of human fibroblasts. Nature.

[10] Hayflick L (1965). The limited in vitro lifetime of human diploid cell strains. Exp Cell Res.

[11] Henson JD, Cao Y, Huschtscha LI, Chang AC, Au AYM, Pickett HA, Reddel RR (2009). DNA C-circles are specific and quantifiable markers of alternative-lengthening-of-telomeres activity. Nat Biotechnol.

[12] Horn S, Figl A, Rachakonda PS, Fischer C, Sucker A, Gast A, Kadel S, Moll I, Nagore E, Hemminki K, Schadendorf D, Kumar R (2013). TERT promoter mutations in familial and sporadic melanoma. Science.

[13] Huang FW, Hodis E, Xu MJ, Kryukov GV, Chin L, Garraway LA (2013). Highly recurrent TERT promoter mutations in human melanoma. Science.

[14] Johnson RA, Wright KD, Poppleton H, Mohankumar KM, Finkelstein D, Pounds SB, Rand V, Leary SE, White E, Eden C, Hogg T, Northcott P, Mack S, Neale G, Wang YD, Coyle B, Atkinson J, DeWire M, Kranenburg TA, Gillespie Y, Allen JC, Merchant T, Boop FA, Sanford RA, Gajjar A, Ellison DW, Taylor MD, Grundy RG, Gilbertson RJ (2010). Cross-species genomics matches driver mutations and cell compartments to model ependymoma. Nature.

[15] Khuong-Quang DA, Buczkowicz P, Rakopoulos P, Liu XY, Fontebasso AM, Bouffet E, Bartels U, Albrecht S, Schwartzentruber J, Letourneau L, Bourgey M, Bourque G, Montpetit A, Bourret G, Lepage P, Fleming A, Lichter P, Kool M, von Deimling A, Sturm D, Korshunov A, Faury D, Jones DT, Majewski J, Pfister SM, Jabado N, Hawkins C (2012). K27M mutation in histone H3.3 defines clinically and biologically distinct subgroups of pediatric diffuse intrinsic pontine gliomas. Acta Neuropathol.

[16] Kim NW, Piatyszek MA, Prowse KR, Harley CB, West MD, Ho PL, Coviello GM, Wright WE, Weinrich SL, Shay JW (1994). Specific association of human telomerase activity with immortal cells and cancer. Science.

[17] Korshunov A, Witt H, Hielscher T, Benner A, Remke M, Ryzhova M, Milde T, Bender S, Wittmann A, Schöttler A, Kulozik AE, Witt O, von Deimling A, Lichter P, Pfister S (2010). Molecular staging of intracranial ependymoma in children and adults. J Clin Oncol.

[18] Mack SC, Witt H, Piro RM, Gu L, Zuyderduyn S, Stütz AM, Wang X, Gallo M, Garzia L, Zayne K, Zhang X, Ramaswamy V, Jäger N, Jones DTW, Sill M, Pugh TJ, Ryzhova M, Wani KM, Shih DJH, Head R, Remke M, Bailey SD, Zichner T, Faria CC, Barszczyk M, Stark S, Seker-Cin H, Hutter S, Johann P, Bender S, Hovestadt V, Tzaridis T, Dubuc AM, Northcott PA, Peacock J, Bertrand KC, Agnihotri S, Cavalli FMG, Clarke I, Nethery-Brokx K, Creasy CL, Verma SK, Koster J, Wu X, Yao Y, Milde T, Sin-Chan P, Zuccaro J, Lau L, Pereira S, Castelo-Branco P, Hirst M, Marra MA, Roberts SS, Fults D, Massimi L, Cho YJ, Van Meter T, Grajkowska W, Lach B, Kulozik AE, von Deimling A, Witt O, Scherer SW, Fan X, Muraszko KM, Kool M, Pomeroy SL, Gupta N, Phillips J, Huang A, Tabori U, Hawkins C, Malkin D, Kongkham PN, Weiss WA, Jabado N, Rutka JT, Bouffet E, Korbel JO, Lupien M, Aldape KD, Bader GD, Eils R, Lichter P, Dirks PB, Pfister SM, Korshunov A, Taylor MD (2014). Epigenomic alterations define lethal CIMP-positive ependymomas of infancy. Nature.

[19] Marchetti A, Pellegrini C, Buttitta F, Falleni M, Romagnoli S, Felicioni L, Barassi F, Salvatore S, Chella A, Angeletti CA, Roncalli M, Coggi G, Bosari S (2002). Prediction of survival in stage I lung carcinoma patients by telomerase function evaluation. Lab Invest.

[20] Marian CO, Cho SK, McEllin BM, Maher EA, Hatanpaa KJ, Madden CJ, Mickey BE, Wright WE, Shay JW, Bachoo RM (2010). The telomerase antagonist, imetelstat, efficiently targets glioblastoma tumor-initiating cells leading to decreased proliferation and tumor growth. Clin Cancer Res.

[21] Meco D, Servidei T, Lamorte G, Binda E, Arena V, Riccardi R (2014). Ependymoma stem cells are highly sensitive to temozolomide in vitro and in orthotopic models. Neuro Oncology.

[22] Mender I, Senturk S, Ozgunes N, Akcali KC, Kletsas D, Gryaznov S, Can A, Shay JW, Dikmen ZG (2013). Imetelstat (a telomerase antagonist) exerts offtarget effects on the cytoskeleton. Int J Oncol.

[23] Mendrzyk F, Korshunov A, Benner A, Toedt G, Pfister S, Radlwimmer B, Lichter P (2006). Identification of gains on 1q and epidermal growth factor receptor overexpression as independent prognostic markers in intracranial ependymoma. Clin Cancer Res.

[24] Milde T, Kleber S, Korshunov A, Witt H, Hielscher T, Koch P, Kopp H-G, Jugold M, Deubzer HE, Oehme I, Lodrini M, Gröne H-J, Benner A, Brüstle O, Gilbertson RJ, Deimling A, Kulozik AE, Pfister SM, Martin-Villalba A, Witt O (2011). A novel human high-risk ependymoma stem cell model reveals the differentiation-inducing potential of the histone deacetylase inhibitor Vorinostat. Acta Neuropathol.

[25] Modena P, Buttarelli FR, Miceli R, Piccinin E, Baldi C, Antonelli M, Morra I, Lauriola L, Di Rocco C, Garrè ML, Sardi I, Genitori L, Maestro R, Gandola L, Facchinetti F, Collini P, Sozzi G, Giangaspero F, Massimino M (2012). Predictors of outcome in an AIEOP series of childhood ependymomas: a multifactorial analysis. Neuro Oncol.

[26] Parker M, Mohankumar KM, Punchihewa C, Weinlich R, Dalton JD, Li Y, Lee R, Tatevossian RG, Phoenix TN, Thiruvenkatam R, White E, Tang B, Orisme W, Gupta K, Rusch M, Chen X, Li Y, Nagahawhatte P, Hedlund E, Finkelstein D, Wu G, Shurtleff S, Easton J, Boggs K, Yergeau D, Vadodaria B, Mulder HL, Becksfort J, Gupta P, Huether R, Ma J, Song G, Gajjar A, Merchant T, Boop F, Smith AA, Ding L, Lu C, Ochoa K, Zhao D, Fulton RS, Fulton LL, Mardis ER, Wilson RK, Downing JR, Green DR, Zhang J, Ellison DW, Gilbertson RJ (2014). C11orf95-RELA fusions drive oncogenic NF-kappaB signalling in ependymoma. Nature.

[27] Pietsch T, Wohlers I, Goschzik T, Dreschmann V, Denkhaus D, Dörner E, Rahmann S, Klein-Hitpass L (2014). Supratentorial ependymomas of childhood carry C11orf95-RELA fusions leading to pathological activation of the NF-κB signaling pathway. Acta Neuropathol.

[28] Remke M, Ramaswamy V, Peacock J, Shih DJH, Koelsche C, Northcott PA, Hill N, Cavalli FMG, Kool M, Wang X, Mack SC, Barszczyk M, Morrissy AS, Wu X, Agnihotri S, Luu B, Jones DTW, Garzia L, Dubuc AM, Zhukova N, Vanner R, Kros JM, French PJ, Van Meir EG, Vibhakar R, Zitterbart K, Chan JA, Bognár L, Klekner A, Lach B, Jung S, Saad AG, Liau LM, Albrecht S, Zollo M, Cooper MK, Thompson RC, Delattre OO, Bourdeaut F, Doz FF, Garami M, Hauser P, Carlotti CG, Van Meter TE, Massimi L, Fults D, Pomeroy SL, Kumabe T, Ra YS, Leonard JR, Elbabaa SK, Mora J, Rubin JB, Cho Y-J, McLendon RE, Bigner DD, Eberhart CG, Fouladi M, Wechsler-Reya RJ, Faria CC, Croul SE, Huang A, Bouffet E, Hawkins CE, Dirks PB, Weiss WA, Schüller U, Pollack IF, Rutkowski S, Meyronet D, Jouvet A, Fèvre-Montange M, Jabado N, Perek-Polnik M, Grajkowska WA, Kim S-K, Rutka JT, Malkin D, Tabori U, Pfister SM, Korshunov A, von Deimling A, Taylor MD (2013). TERT promoter mutations are highly recurrent in SHH subgroup medulloblastoma. Acta Neuropathol.

[29] Ridley L, Rahman R, Brundler MA, Ellison D, Lowe J, Robson K, Prebble E, Luckett I, Gilbertson RJ, Parkes S, Rand V, Coyle B, Grundy RG (2008). Multifactorial analysis of predictors of outcome in pediatric intracranial ependymoma. Neuro Oncol.

[30] Rogers HA, Mayne C, Chapman RJ, Kilday JP, Coyle B, Grundy RG (2013). PI3K pathway activation provides a novel therapeutic target for pediatric ependymoma and is an independent marker of progression-free survival. Clin Cancer Res.

[31] Serrano D, Bleau AM, Fernandez-Garcia I, Fernandez-Marcelo T, Iniesta P, Ortiz-de-Solorzano C, Calvo A (2011). Inhibition of telomerase activity preferentially targets aldehyde dehydrogenase-positive cancer stem-like cells in lung cancer. Mol cancer.

[32] Shim KW, Kim DS, Choi JU (2009). The history of ependymoma management. Childs Nerv Syst.

[33] Singh SK, Clarke ID, Terasaki M, Bonn VE, Hawkins C, Squire J, Dirks PB (2003). Identification of a cancer stem cell in human brain tumors. Cancer Res.

[34] Smyth MD, Horn BN, Russo C, Berger MS (2000). Intracranial ependymomas of childhood: current management strategies. Pediatr Neurosurg.

[35] Sowar K, Straessle J, Donson AM, Handler M, Foreman NK (2006). Predicting which children are at risk for ependymoma relapse. J Neurooncol.

[36] Tabori U, Ma J, Carter M, Zielenska M, Rutka J, Bouffet E, Bartels U, Malkin D, Hawkins C (2006). Human telomere reverse transcriptase expression predicts progression and survival in pediatric intracranial ependymoma. J Clin Oncol.

[37] Tabori U, Vukovic B, Zielenska M, Hawkins C, Braude I, Rutka J, Bouffet E, Squire J, Malkin D (2006). The role of telomere maintenance in the spontaneous growth arrest of pediatric low-grade gliomas. Neoplasia.

[38] Vaidya K, Smee R, Williams JR (2012). Prognostic factors and treatment options for paediatric ependymomas. J Clin Neurosci.

[39] Vinchon M, Leblond P, Noudel R, Dhellemmes P (2005). Intracranial ependymomas in childhood: recurrence, reoperation, and outcome. Childs Nerv Syst.

[40] Wani K, Armstrong TS, Vera-Bolanos E, Raghunathan A, Ellison D, Gilbertson R, Vaillant B, Goldman S, Packer RJ, Fouladi M, Pollack I, Mikkelsen T, Prados M, Omuro A, Soffietti R, Ledoux A, Wilson C, Long L, Gilbert MR, Aldape K, Collaborative Ependymoma Research N (2012). A prognostic gene expression signature in infratentorial ependymoma. Acta Neuropathol.

[41] Wong VCH, Ma J, Hawkins CE (2009). Telomerase inhibition induces acute ATM-dependent growth arrest in human astrocytomas. Cancer Lett.

[42] Wong VCH, Morrison A, Tabori U, Hawkins CE (2010). Telomerase inhibition as a novel therapy for pediatric ependymoma. Brain Pathol.

[43] Wright WE, Piatyszek MA, Rainey WE, Byrd W, Shay JW (1996). Telomerase activity in human germline and embryonic tissues and cells. Dev Genet.

[44] Wu YL, Dudognon C, Nguyen E, Hillion J, Pendino F, Tarkanyi I, Aradi J, Lanotte M, Tong JH, Chen GQ, Ségal-Bendirdjian E (2006). Immunodetection of human telomerase reverse-transcriptase (hTERT) re-appraised: nucleolin and telomerase cross paths. J Cell Sci.

[45] Yu L, Baxter PA, Voicu H, Gurusiddappa S, Zhao Y, Adesina A, Man TK, Shu Q, Zhang YJ, Zhao XM, Su JM, Perlaky L, Dauser R, Chintagumpala M, Lau CC, Blaney SM, Rao PH, Leung H-CE, Li X-N (2010). A clinically relevant orthotopic xenograft model of ependymoma that maintains the genomic signature of the primary tumor and preserves cancer stem cells in vivo. Neuro Oncol.

[46] Zacharoulis S, Moreno L (2009). Ependymoma: an update. J Child Neurol.

