# Supplementary material for: Telomerase inhibition abolishes the tumorigenicity of pediatric ependymoma tumor-initiating cells
Source: Acta Neuropathol. 2014 Aug 6;128(6):863–77. doi: 10.1007/s00401-014-1327-6 (PMC4286630; doi:10.1007/s00401-014-1327-6)
Supplement: Supplementary file 7 — Supplementary material 7 (DOCX 36 kb) [file 401_2014_1327_MOESM7_ESM.docx]

Table S2: Multivariate Survival Analysis of Pediatric Ependymoma Cohort

| **Factor** | **Hazard Ratio** | **Significance** |
| --- | --- | --- |
| Age at Diagnosis (>3 yrs) | 1.84 | 0.42 |
| Location | 0.85 | 0.84 |
| Extent of surgical resection | 1.88 | 0.11 |
| Telomerase Activity | 5.67 | 0.10 |
