# Supplementary material for: Telomerase inhibition abolishes the tumorigenicity of pediatric ependymoma tumor-initiating cells
Source: Acta Neuropathol. 2014 Aug 6;128(6):863–77. doi: 10.1007/s00401-014-1327-6 (PMC4286630; doi:10.1007/s00401-014-1327-6)
Supplement: Supplementary file 8 — Supplementary material 8 (DOCX 52 kb) [file 401_2014_1327_MOESM8_ESM.docx]

Table S3: Characterization of Pediatric Ependymoma Cell Models

| **Characteristic** | **BXD** | **R254** | **E520** |
| --- | --- | --- | --- |
| Location | Supratentorial | Supratentorial | Infratentorial |
| hTERT promoter mutation | - | + | + |
| hTERT promoter hypermethylation | + | + | + |
| Telomerase activity | + | + | + |
| *C11orf95-RELA* fusion | + | + | n/a |
| CIMP status | n/a | n/a | + |
| Copy number aberrations | 1q gain (incl. 1q25), 6q loss, *CDKN2A* loss | 1q gain (incl. 1q25), 1p gain (incl. *EPHB2*), 9q gain (incl. *TNC*, *NOTCH1*), *CDKN2B* loss, *PTEN* loss | n/a |
